# Acute histoplasmosis in immunocompetent travelers: a systematic review of literature

**DOI:** 10.1186/s12879-018-3476-z

**Published:** 2018-12-18

**Authors:** Silvia Staffolani, Dora Buonfrate, Andrea Angheben, Federico Gobbi, Giovanni Giorli, Massimo Guerriero, Zeno Bisoffi, Francesco Barchiesi

**Affiliations:** 1 Centro per le Malattie Tropicali, IRCCS Istituto di Ricovero e Cura a Carattere Scientifico, Ospedale Sacro Cuore – Don Calabria, Via Don Sempreboni 5, 37024 Verona, Negrar Italy; 2 SOD Malattie Infettive emergenti e degli immunodepressi, Azienda Ospedaliero Universitaria, Ospedali Riuniti di Ancona, Via Conca Torrette, Ancona, Italy; 3 Computer Sciences, Dipartimento di Economia Aziendale, Università degli Studi di Verona, Strada le Grazie, Verona, Italy; 4 Sezione di Malattie Infettive e Tropicali, Dipartimento di Diagnostica e Sanità Pubblica, Università di Verona, Strada le Grazie, Verona, Italy; 5 Clinica Malattie Infettive, Dipartimento di Scienze Biomediche e Sanità Pubblica, Università Politecnica delle Marche, Azienda Ospedaliera Umberto I° Via Conca Torrette, Ancona, Italy

**Keywords:** Histoplasma, Histoplasmosis, Acute, Travel, Immunocompetent

## Abstract

**Background:**

Histoplasmosis is a fungal infection highly endemic in the American continent. The disease can be severe in immunocompromised subjects. In immunocompetent subjects the clinical manifestations are variable. Aim of this work was to review the cases of acute histoplasmosis in immunocompetent travelers reported in literature.

**Methods:**

A systematic review of literature was conducted. Electronic search was performed in Pubmed and LILACS. Two reviewers independently extracted data on demographic, clinical and radiological features, and treatment. Cases were classified according to Wheat’s definitions.

**Results:**

Seventy-one studies were included in the analysis, comprising a total of 814 patients. Twenty-one patients diagnosed at the Centre of Tropical Diseases, Negrar (VR), Italy were also included. The most common travel destination was Central America (168 people, 29.8%); the most common way of exposure to histoplasma was the exploration of caves and/or contact with bat guano (349 people, 60.9%). The multivariate logistic regression model showed association between the development of disseminated histoplasmosis (DH) and activities that involved the exploration of caves and/or the contact with bats’ guano (adjusted OR: 34.20 95% CI: 5.29 to 220.93) or other outdoor activities (adjusted OR: 4.61 95% CI: 1.09 to 19.56). No significant difference in the attack rate between countries of destination was observed (*p*-value: 0.8906, Kruskal-Wallis test).

**Conclusions:**

Histoplasmosis often causes no or mild symptoms in immunocompetent individuals, although a severe syndrome may occur. The infection can mimic other diseases, and the epidemiological risk of exposure is an important clue to raise the index of suspicion.

**Electronic supplementary material:**

The online version of this article (10.1186/s12879-018-3476-z) contains supplementary material, which is available to authorized users.

## Background

Histoplasmosis is a fungal infection acquired by inhalation of *Histoplasma capsulatum* var. *capsulatum* microconidia. This fungus is endemic in the American continent (North, Central and South America). Cases have also been described in Africa, Asia, and Europe [1].

Histoplasmosis is both a disease originating from soil (infections often occur after human disruption of the soil that aerosolizes the hyphae and conidia of the organism originating from the earth enriched with birds’ excreta) and a sapronosis (originating from bats) [2, 3]. With rare exceptions, this fungus infects.

its host via the respiratory route: microconidia are inhalated and deposited in the terminal bronchioles and alveoli of the lung.

Clinical manifestations of histoplasmosis depend on the size of the inoculum and the patient’s immunological status/underlying conditions. Low level of exposure in the healthy host typically leads to an asymptomatic infection, while the acute syndrome usually follows a heavy inoculum [4]. In the latter case, chest radiographs usually show diffuse interstitial or reticulonodular infiltrates, sometimes associated with hilar or mediastinal lymphadenopathy [4]. Systemic symptoms include fever with possible respiratory impairment, sweats, weight loss, headache and gastrointestinal complaints. A subacute pulmonary syndrome is also possible in case of low inoculum [4]. The most frequent symptoms are cough, fever, malaise and fatigue, typically occurring over weeks to months before seeking medical advice [4]. Solitary lung nodules (histoplasmomas) or multiple pulmonary nodules may develop, characterized by indefinite persistence of foci of infection.

Calcification is usually found in the center of a histoplasmoma or in concentric rings and is generally diagnostic, although it may require years to develop and is not always present.

Pulmonary nodules may slowly enlarge and even cavitate [5]; moreover, they are usually identified as incidental findings on chest radiographs or CT scans, and differential diagnosis includes sarcoidosis, tuberculosis (TB) and malignancies [6–8]. Chronic pulmonary histoplasmosis can be observed in individuals with underlying lung conditions, and it is defined as failure to clear *H. capsulatum* infection [4].

Symptoms and signs include cough, low-grade fever, night sweats and weight loss. Chest radiographs show interstitial or consolidative infiltrates associated with cavitation or fluid-filled emphysematous bullae [4]. This kind of pulmonary syndrome doesn’t resolve spontaneously, leading to gradual progression and destruction of lung parenchyma. Mediastinitis, broncholitiasis, pericarditis and a rheumatologic syndromes have been described, too. The disease is particularly relevant for immunosuppressed subjects, who are at risk of developing the disseminated form, which is often fatal if not promptly recognized [4].

On the other hand, the infection is usually mild and self-limiting in immunocompetent people [4], although, apparently in rare cases, the disease can progress to a severe form with high morbidity even in those individuals [9–12].

### Objectives

Aim of our work was to review the literature on acute histoplasmosis in immunocompetent travelers, describing the main characteristics of the infection in this subgroup of patients. Secondary objectives were to investigate the characteristics associated to the development of disseminated histoplasmosis in travelers, and to estimate the attack rate in different areas of the world.

## Methods

We conducted a systematic review of case reports and case series available in literature. We analyzed the epidemiological and clinical features, diagnostic work-up, treatment, and outcome of cases of acute histoplasmosis in immunocompetent patients, with history of recent travel (≤ 3 months) in an endemic area.

Electronic search was conducted in PubMed and LILACS on 31st December 2016, and all papers published up to that date were included. We included full texts written in English, Italian, French, German, Spanish, Portuguese.

Additional cases were sought from the reference list of included papers and of non-included reviews. Moreover, grey literature was sought through contact with authors. Two authors, SS and AA, independently screened the list of papers generated from the electronic search, using EndNote program, version 6, 2012 Thomson Reuters.

SS and AA extracted the data on the basis of inclusion/exclusion criteria and case definitions (reported below), and entered all information in a database created with Microsoft Office Excel 2007. Data collected were: age, sex, country of exposure, activities done in the country, attack rate and development of disseminated disease. The Excel data base is available as supplementary material (Additional file 1).

In case of discrepancies in the process of inclusion of papers/data extraction, a consensus was reached through discussion or involvement of a third reviewer (FG).

### Inclusion criteria

-Case reports or case series on acute histoplasmosis.

-Patients with history of travel in an endemic area (including travels from an area of no/low endemicity to an area of high endemicity within a country) ≤ 3 months before the onset of symptoms.

-Immunocompetence status.

### Exclusion criteria

-Reviews of literature.

-Cases describing patients presenting with radiologic findings compatible with histoplasmosis (i.e. pulmonary nodules) but no apparent temporal relationship with travel.

-Cases native from an endemic area without history of travel to an area with higher endemicity.

-Non-immunocompetence status at the time of the travel, at the onset of symptoms or at the time of diagnosis of histoplasmosis.

-Occupational exposure in native area.

-Cases of reactivation of histoplasmosis acquired in the past.

-Cases of histoplasmosis due to *Histoplasma capsulatum* var. *duboisii*.

### Case definition (adapted from wheat [13] and EORTC/MSG criteria [14])

#### Proven

Illness consistent with acute histoplasmosis in a patient who had recently (≤ 3 months) travelled to an endemic area and one of the following:

-Positive culture from a specimen obtained from the affected site or tissue.

-Histopathologic or direct microscopic demonstration of yeast intracellular forms or yeasts in tissue macrophages.

#### Probable

-Host factor: recent travel (≤ 3 months) in area endemic for histoplasmosis.

-Clinical picture and/or radiological findings consistent with acute histoplasmosis.

-Mycological evidence (histoplasmin test positivity and/or positivity of serology and/or positivity of urine-serum *Histoplasma* antigen).

#### Possible

-Host factor: recent travel (≤ 3 months) in area endemic for histoplasmosis.

-Clinical picture and/or radiological findings consistent with acute histoplasmosis.

-No mycological evidence nor serologic evidence but:

Belonging to a histoplasmosis cluster and/or efficacy of oral ex juvantibus itraconazole therapy (resolution of symptoms and normalization of radiological findings).

### Activities at risk for developing histoplasmosis

Contact with bats excreta and/or visit to a cave.

Outdoor activities: forest excursions/trekking, camping or other activities not involving visit to caves or direct contact with bats’ excreta.

Activities involving contact with chickens or other non-mammal birds.

All activities that put the subject at risk of inhalating *Histoplasma* microconidia: building construction, renovation, excavation (biologists, building contractors, construction workers, soldiers, volunteers involved in churches or other buildings renovation).

### Syndromic classification (according to wheat [13])

Asymptomatic (As): asymptomatic case identified through laboratory screening within a histoplasmosis cluster.

Acute pulmonary histoplasmosis (AP): febrile illness with respiratory symptoms and/or mediastinal involvement and/or accompanying constitutional symptoms and/or gastrointestinal or cutaneous involvement when they are not the main manifestation.

Rheumathologic (R): a case with arthralgia or arthritis as unique symptom.

Disseminated Histoplasmosis (DH): febrile illness with demonstration of bone marrow involvement (leucopenia, thrombocytopenia, anemia) AND/OR disseminated monocytic-macrophage involvement (hepatosplenomegaly AND/OR increased liver enzymes), with or without respiratory symptoms.

### Statistical analysis

Descriptive statistics were used to analyze the characteristics of the entire cohort. Categorical variables were reported as frequencies and proportions, while quantitative variables were presented as median (interquartile range, IQR). Associations between covariates were explored through univariate logistic models and non-parametric Kruskal-Wallis rank sum test. Lastly, we fitted a multivariate logistic regression model to assess the impact of all covariates on the probability of contracting the disseminated disease. All statistical analyses were conducted using R, version 3.3.3 [15].

## Results

**Fig. 1:**
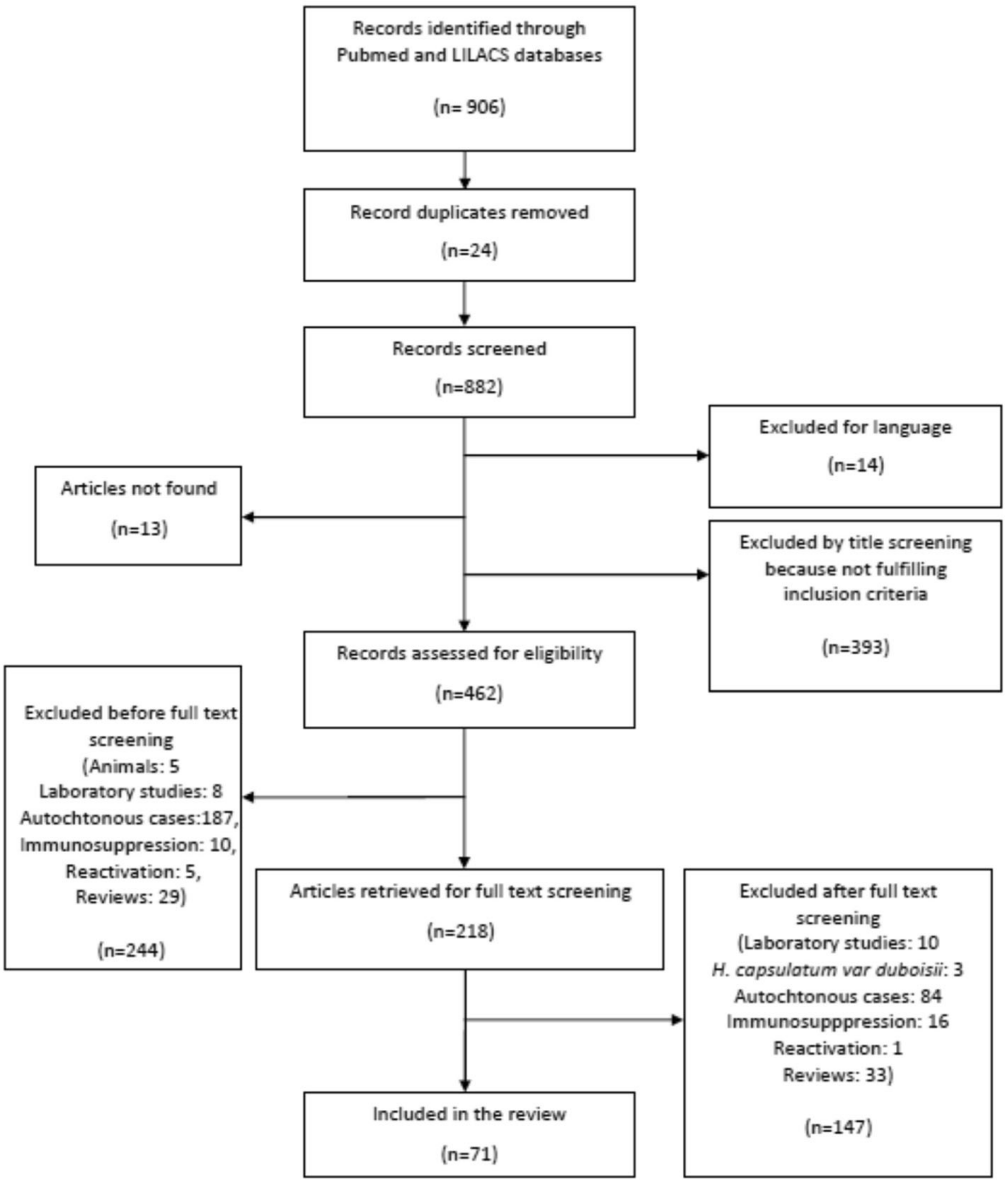
PRISMA flow chart for study selection

**Fig. 2:**
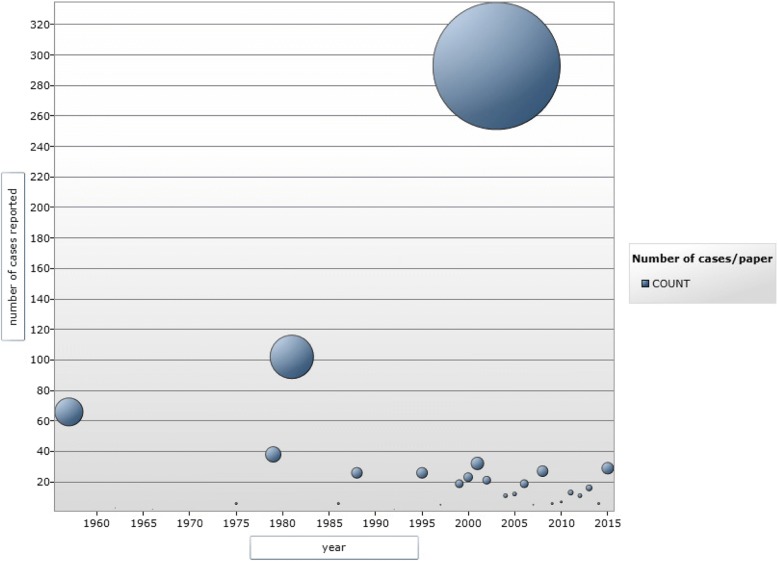
Timeline distribution of the studies reporting cases of acute histoplasmosis in immunocompetent travelers. Each bubble corresponds to a study, the diameter of the bubble is proportional to the number of cases described in the study, which correspond to the projection of the center of the bubble on the ordinate axis

**Fig. 3:**
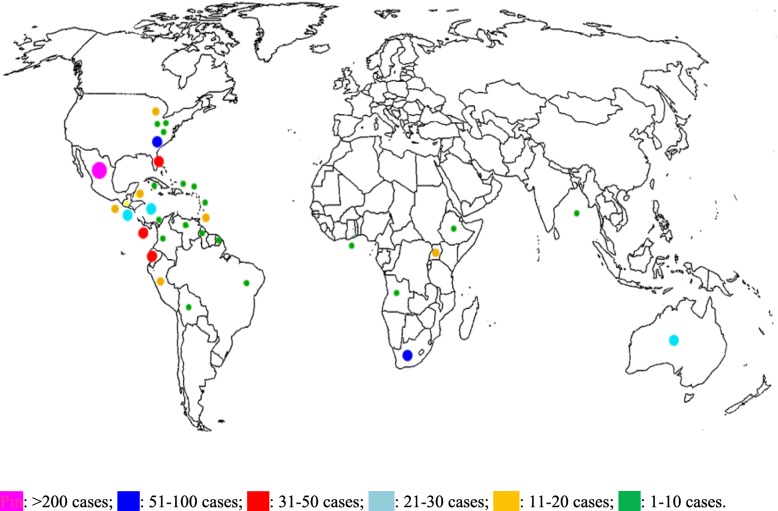
Distribution of acute histoplasmosis reported cases among immunocompetent travelers

The study selection process (PRISMA flow chart) is shown in Fig. 1. The studies included in the analysis through electronic search were 71 [8, 16–85], (Table 1) comprising a total of 814 patients (see Fig. 2 for the timeline distribution of studies and cases respectively). In the analysis were also included 21 patients diagnosed at the Centre of Tropical Diseases (CTD) of Negrar, Verona, Italy during the decade 2005–2015 (Staffolani S, Buonfrate D, Farina C, Gulletta M, Gobbi F, Angheben A, manuscript in preparation), for a total of 835 patients. Figure 3 shows the countries of presumable exposure to the infection.

**Table 1:** Studies selected for the systematic review

	Reference	Publication year	N° cases	Country of origin	Visited continent	Visited country	Reason of travel	Exposure	Cluster	Syndromic classification	Therapy	Type of therapy
1	[8]	2015	1	Swiss	SA	Brazil, Argentina		C	no	AP	yes	itra
2	[16]	1988	15	NS	CA	Costa rica	ST	B	yes	AP	no	
3	[17]	2007	4	Spain	SA	Ecuador	V	C, S	yes	AP	no	
4	[18]	2004	1	France	SA	Colombia	T	B	no	AP	yes	NS
5	[19]	2011	2	Italy	SA	Ecuador		O	no	AP, DH	1 yes	Itra
6	[20]	1992	1	Germany	SA	Ecuador	T	O	no	AP	no	
7	[21]	2013	1	Israel	SA, A	Jordan, Bolivia, Brazil, Ethiopia, Angola	T	O	no	DH	yes	Itra
8	[22]	1995	2	France	SA	Guyana	P	O, S	no	AP	yes	Excision
9	[23]	2011	5	Spain	A, SA, CA	Angola, Venezuela, Nicaragua, Ecuador	T, P, SP, V	B, O	no	AP	no	
10	[24]	2002	14	Canada	CA	Belize	ST	O	yes	AP	1 yes	itra
11	[25]	2013	1	USA	SA	Peru	V	B, S	no	DH	yes	AmB, itra
12	[26]	1999	1	France	SA	Guyana	P	O, S	no	DH	yes	itra
13	[27]	2013	12	Europe, Africa	A	Uganda	ST	O	yes	AP	5 yes	4 itra, 1 keto
.14	[28]	1979	10		A	South Africa	SP	B	yes	9 AP, 1 DH	1 yes	AmB
15	[29]	1997	1	France	SA	Guyana	P	O, S	no	AP	yes	Itra
16	[30]	2006	1	Netherland	A	Ghana	T	B, O	no	AP	no	
17	[31]	1957	2	South africa	A	South Africa	SP	B	yes	AP	no	
18	[32]	2012	1	Germany	SA	Costa Rica	T	B, O	no	AP	yes	itra
19	[33]	2002	6	Germany	CA	Cuba	BIO	B, S	yes	3 AP, 3 DH	yes	itra
20	[34]	2010	3	Japan		Malaysia	T	O	yes	AP	no	
21	[35]	2003	262	USA		Mexico	T	O, S	yes	AP	yes	NK
22	[36]	2000	4	Italy	CA, SA	Guatemala, Dominican republic, Peru	T, SP	B, O, S	no	3 AP, 1 DH	yes	2keto, 1 itra, 1 amB
23	[37]	2005	1	Italy	CA	Nicaragua	P	O, S	no	AP	yes	itra
24	[38]	2003	3	Spain	CA	Nicaragua	T	B, O	yes	AP	1 yes	itra
25	[39]	1988	11	USA	USA	Iowa	T	O, S	yes	AP	no	
26	[40]	2005	9	Spain	SA	Guatemala	V	S	yes	AP	no	
27	[41]	2000	7	Spain	CA, SA	Dominican Republic, Nicaragua, Colombia, Peru, Guatemala	T, V	B, O, S	no	1 AP, 8 DH	yes	itra
28	[42]	1962	3	Europe	A	South Africa	SP	B	yes	DH	yes	AmB
29	[43]	1990	1	France	SA	Guyana	T	O	no	AP	no	
30	[44]	1981	69	USA	NA	South Carolina	CI	C	yes	AP	no	
31	[45]	1975	6	Canada	CA	Puerto Rico	ST, TEA	B, O	yes	AP	no	
32	[46]	1979	1	Jamaica	CA	Caribbean	TEA	B	no	AP	no	
33	[47]	1996	1	Germany	CA		T	O	no	AP	no	
34	[48]	2008	3	Austria		Mexico	T	B	no	DH	yes	itra
35	[49]	2008	3	Germany	CA	Antille	BIO	B, S	yes	2 DH, 1 AP	yes	itra
36	[50]	2012	4	Poland	SA	Ecuador	T	B	yes	DH	2 yes	keto
37	[51]	2011	2	France	CA	Costa Rica	T	O	yes	AP	1 yes	1 itra
38	[52]	2003	1	Germany		Mexico, Brazil	T	O	no	AP	no	
39	[53]	1979	27	USA	USA	North-Centre Florida	V	B, O, S	yes	8 As, 2 DH 17 AP	2 yes	2 AmB
40	[54]	2004	10	USA	CA	Costa Rica	T	B	yes	AP	no	
41	[55]	2013	1	Canada	CA	Costa Rica		B, O, S	no	DH	yes	AmB, itra
42	[56]	2005	2	France	SA	Venezuela	P	O, S	no	AP	yes	itra
43	[57]	1991	1	France	SA	Guatemala	T	O, S	no	AP	yes	keto
44	[58]	2014	1	Germany	SA	Ecuador	T	O	no	AP	yes	itra
45	[59]	1957	64	Europe, NS	A	South Africa	ST, SP	B	yes	AP, 5 As	no	
46	[60]	1997	4	Italy	SA	Peru	SP	B, C	yes	DH	yes	keto
47	[61]	1995	24	Europe, SEA	AU	New Caledonia	SP	B	yes	AP, 3 As	21 yes	Keto, itra, AmB
48	[62]	2009	5	Spain	CA, SA	Peru, Costa Rica, El Salvador, Panama, Ecuador	T, BIO, V, P	B, C, O, S	no	3 AP, 2 DH	Yes 1 pulmonary and 2 DH	itra
49	[63]	2001	1	Spain	SA	Peru	T	B, O	no	AP	yes	itra
50	[64]	2014	4	Brazil	SA	Brazil	BIO	B	yes	AP	2 yes	itra
51	[65]	1986	6	USA	USA	Florida	ST, P	B, S	yes	AP	4 yes	2Amb,2keto
52	[66]	2006	1	USA	CA	Guatemala	T	O	no	R	yes	itra
53	[67]	2003	13	France	CA	Martinica	Trekking trip	O, S	yes	11 DH, 2 AP	yes	itra
54	[68]	2009	1	Germany	CA	Antille	BIO	B, S	no	AP	yes	NS
55	[69]	2015	23	Israel	USA, CA, SA	Guatemala, Costa Rica Peru, Mexico, Bolivia, Indiana, Dominican Republic	NS	B, O	no	14 AP, 9 As	Not known	
56	[70]	2011	3	France	CA	Cuba	SP	B	yes	1 DH, 2 AP	yes	itra
57	[71]	2007	1	Japan	SA	Bolivia	P	O, S	no	AP	yes	fluco
58	[72]	1992	1	Italy	A		P	O, S	no	AP	yes	AmB
59	[73]	2000	12	Spain	CA, SA	Guatemala, Honduras, Nicaragua, Dominican Republic, Peru	V, SP, T	B, O, S	yes	7 AP, 5 As	8 yes	itra
60	[74]	1999	11	USA	SA	Ecuador	ST	B	yes	1 DH, 8 AP, 2 not possible	4 yes	1 itra, 3 systemic azoles
61	[75]	2008	20	USA	CA	El Salvador	V	O, S	yes	AP	Not known	
62	[76]	2003	14	USA	CA	Nicaragua	ST	B	yes	4 DH, 8 AP, 2 As	yes	itra
63	[77]	1999	6	Chile	SA	Ecuador	T	C, O	yes	AP	yes	itra
64	[78]	1966	2	USA	USA	Missouri, Kentucky	T	C	no	AP	1 yes	AmB
65	[79]	2015	1	USA	USA	Southwest	T	O, S	no	AP	yes	itra
66	[80]	2010	4	Brasil	SA	Brasil	SP	B	yes	2 AP, 2 DH	yes	
67	[81]	2012	5	Argentina	SA	Argentina	O	C	Yes	AP	No	
68	[82]	2002	1	Chile	SA	Peru	SP	B	No	AP	Yes	
69	[83]	2001	31	Venezuela	SA	Venezuela	SP	B	Yes	AP	No	
70	[84]	1981	33	colombia	SA	colombia	SP	B	yes	AP	no	
71	[85]	1999	1	Taiwan	SEA	Indonesia	P	O	no	DH	yes	AmB
CTD Negrar		2016	21	Italy	SA, CA	Ecuador, Bolivia, Mexico, Cuba	T, SP, P	B, O, S	16 yes, 5 no	1 DH, 20 AP	11 yes	itra

*NS* not specified, *USA* United States of America, *NA* North America, *CA* Central America, *SA* South America, *A* Africa, *AU* Australia, *SEA* South East Asia, *ST* student, *V* Volunteer, *T* Tourist, *P* professional, *SP* Speleologist, *BIO* Biologist, *CI* Correctional Institute *TEA* Teacher, *B* Cave_bats, *C* Birds/chickens, *S* Moved Soil, *O* Outdoor activities, *DH* Disseminated Histoplasmosis, *AP* Acute Pulmonary Histoplasmosis, *AmB* Amphotericin B, *Itra* Itraconazole, *Fluco* Fluconazole, *Keto* Ketoconazole

Regarding the cases diagnosed at the Centre of Tropical Diseases, Negrar (VR), briefly, we found 21 cases of acute histoplasmosis in immunocompetent subjects, 17 of them affecting members of a scientific expedition to Ecuador. The other 4 patients did not belong to a cluster and traveled to Panama, Bolivia, Mexico, Cuba respectively. Seventeen out of 21 patients were male (81%). The mean age was 38.5 years for the Ecuador cluster, 46.7 years for the other cases. They were infected through contact with bat excreta, inhalation of contaminated soil, outdoor activities. All cases were classified as possible histoplasmosis, but two probable for positive serology and two proven cases based on histology. The main symptoms were fever (12/21, 57%), respiratory (12/21, 57%) and gastrointestinal (9/21, 43%) symptoms. Seven patients (33%) were admitted to the hospital and four of them underwent invasive diagnostic procedures (bronchoscopy, biopsy of other tissues). Eleven out of 17 (65%) patients of the Ecuador cluster and 4/4 (100%) patients not belonging to it had abnormal blood exams. Eleven patients (65%, 7/17 (41%) in the cluster and 4/4 (100%) not belonging to the Ecuador cluster) received oral itraconazole. Nine patients (39.1%) had persistent lung nodules at the 12-month radiological follow up.

Regarding all selected cases of the present review, most patients (488/835, 59%) belonged to a cluster.

There were 45 clusters described by 36 authors, plus 2 clusters diagnosed at CTD. The cluster comprising the largest number of individuals (*n* = 262) was described by Morgan [35].

While we considered all 835 patients for the description of main characteristics of cases, the “Acapulco cluster” [35] was removed from our dataset for univariate and multivariate analyses, as it did not contain information regarding age or the state of disseminated disease. Table 2 displays a summary of the characteristics of the cohort of the 573 immunocompetent travelers considered for univariate and multivariate analyses.

**Table 2:** Baseline Demographic and Travel Characteristics of the Entire Cohort of Immunocompetent Travelers

Characteristic	Entire Cohort
	N, %
Total	573
Median age, years (IQR)	29 (23–43)
Male, N (%)	244 (64.0)
Median AR ^a^, % (IQR)	90 (73.2–100.0)
Continent of destination, N (%)	
USA-Canada	117 (20.8)
Central America	168 (29.8)
South America	154 (27.4)
Africa	95 (16.9)
Others ^b^	29 (5.1)
Activity ^c^, N (%)	
Caves/Bat guano	349 (60.9)
Outdoor activities	198 (34.5)
Bird_chickens	105 (18.3)
Inhalation_soil	104 (18.1)

*Abbreviations, AR* attack rate, *IQR* interquartile range

^a^ Calculated as the proportion of infected people in the travel group. We identified 47 travel groups in the cohort

^b^ Includes: Asia, Oceania

^c^ Frequencies exceed 100%, as most people did more than one activity during the trip. Caves/Bat’s guano: contact with bats excreta and/or visit to a cave. Outdoor activities: forest excursions/trekking, camping or other activities not involving visit to caves or direct contact with bats’ excreta. Bird_chickens: activities involving contact with chickens or other non-mammal birds. Inhalation_soil: all activities involving building construction, renovation, excavation (biologists, building contractors, construction workers, soldiers, volunteers involved in churches or other buildings renovation)

### Clinical-syndromic classification

According to the above-mentioned definitions, we found 32 proven, 695 probable and 108 possible histoplasmosis cases.

Most patients (605/724, 84%) had acute pulmonary histoplasmosis (AP). Of them, 111 (18%) had also rheumatologic manifestations and 26 (4%) mediastinal involvement. In this group we also included patients with miliary pulmonary lesions if they had only respiratory symptoms with no signs of dissemination. Fifty-six (8%) cases were classified as Disseminated Histoplasmosis (DH), 65 (9%) patients were asymptomatic (A), 9 (1%) patients had only rheumatologic (R) manifestations.

### Symptoms

**Fig. 4:**
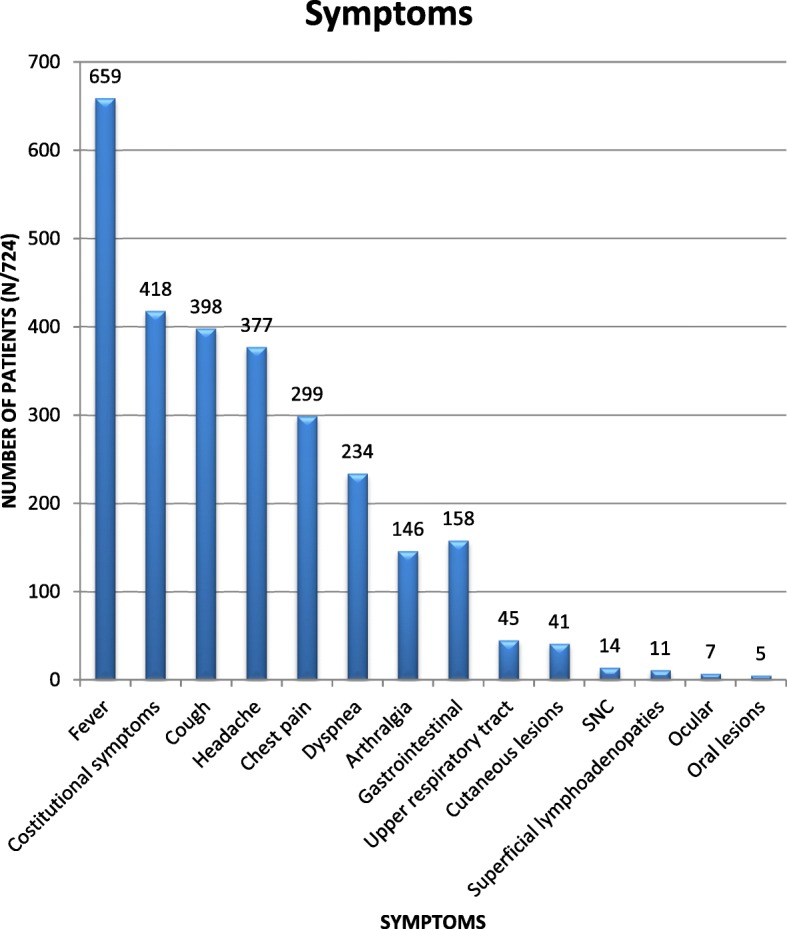
Frequency of symptoms reported

This information was available for 789/835 (94.5%) subjects, of whom 724 reported symptoms. As it is shown in Fig.4, the most frequent symptoms were fever (659/724, 91%), cough (398, 55%), headache (377, 52%) and chest pain (299, 41%). Constitutional symptoms (myalgia and/or sweats, and/or weight loss, and/or anorexia) were reported by 418 (58%) patients. Patients reporting chills, with or without documented fever, were 91 (13%). Gastrointestinal symptoms (158/724, 22%) comprised diarrhea, nausea, abdominal pain or vomit. Oral lesions (5/724, 0.7%) were aftous in 3 cases, ulcerative (lingual and tonsillar), and papular (lingual) in one patient each. Skin abnormalities (41/724, 5.5%) were: diffuse macular rash in 32/41 (78%) patients, erythema nodosum in 5 (12%), while cutaneous ulcers, papules, and splinter hemorrhages were reported in one case each. For a further patient the type of cutaneous lesion was not specified. Symptoms attributed to central nervous system impairment (14/724, 2%) included irritability, insomnia, dizziness, lethargy.

Ophtalmological symptoms (7/724, 1%) were conjunctivitis, ocular pain, photofobia.

### Laboratory exams

This information was available for 212 (25%) patients. Of them, 90 (42%) had abnormal laboratory exams (abnormal leucocyte and/or platelet count or anemia, increased inflammatory markers, increased LDH, abnormal liver enzymes values). In particular: 27 (30%) had abnormal leucocyte and/or platelet count or anemia, 62 (69%) had increased inflammatory markers, 26 (29%) had increased LDH, 45 (50%) had abnormal liver enzymes values.

Information about *Histoplasma* serology was available for 634 (76%) patients, of whom 413 (65%) were positive. All asymptomatic patients included in the analysis were positive to serology, which was performed on the basis of epidemiological suspicion (travel with patients diagnosed with histoplasmosis). Of 213 (26%) patients screened with histoplasmin skin test, 199 (93%) resulted positive.

### Imaging

Imaging included chest X ray and CT scan. This information was available for 376 (45%) patients. Of them, 250 (67%) showed abnormal imaging. In particular: nodular infiltrates were present in 200 patients (80%), patches in 16 patients (6%), miliary pattern in 14 patients (6%), hylar/mediastinal adenopathies in 49 patients (20%), pleural effusion in 4 patients (2%). Information about the spleen and liver size was available only for 14 (2%) patients, of whom 5 (36%) had hepatosplenomegaly and one had hepatomegaly.

### Hospitalization and invasive procedures

Information about hospitalization was available for 595 (71%) patients, of whom 130 (22%) were admitted to hospital. Information about diagnostic procedures was available for 742 patients (89%). Of them, 64 patients (9%) underwent invasive procedures. Usually, obtained specimens were sent for coltural, cytologic and/or histological examination. Fiftyseven out of 62 patients (92%) had a positive colture for *Histoplasma*, 9/61 (15%) had positive cytologic examination (7 of them had a positive hystologic exam, too), 37/63 (59%) had positive pathology findings (of them 21 had also positive colture and 4 had also a positive cytologic examination).

Fourtyfive patients (70%) underwent bronchoscopy with bronchoalveolar lavage (BAL), and 21 (47%) of them underwent lymphonodal biopsy, too. Of all them, 26/44 (59%) had positive mycologic colture, 8/44 (18%) had positive cytologic exam and 20/45 (44%) had positive histologic exam. Eighteen patients had more than one positive result.

Thirteen patients (20%) were submitted to transthoracic lung biopsy. Seven out of 13 (54%) coltural exams, 1/13 (8%) cytologic exams and 9/13 (69%) histological exams were positive. Four patients had more than one positive exam.

Eight patients (13%) underwent lung resection. Two out of 8 (25%) coltural and 7/7 (100%) histological examinations were positive.

Finally, six patients (9%) underwent biopsies of tissues other than lung (bone marrow 5 patients, skin one patient). Five out of 6 (83%) coltural exams and 5/6 (83%) histological exams were positive. Four patients had both coltural and histological positive exams.

### Treatment

Information was available for 744 patients (89%). Of them, 185 (25%) received antifungal therapy. In particular: 105 (57%) patients received oral itraconazole, while 21 (11%) received intravenous amphotericin B. Twelve (57%) of the latter were switched to oral drugs at hospital discharge: one of them was switched initially to oral itraconazole and then to posaconazole, 8 of them were switched to ketoconazole and then to itraconazole, 4 were switched to itraconazole. Twenty-four (13%) patients were treated with systemic azoles other than itraconazole, while 33 (18%) patients were treated with an unspecified antifungal therapy. A couple of patients with lung nodules underwent excisional surgery and no antifungal therapy.

### Outcome

A detailed information about the outcome was available for 727 (87%) patients. Most of them (726/727, 99.8%) recovered. In particular: 688 (95%) patients had complete clinical and radiological resolution at the last visit, while 38 (5%) patients had clinical recovery and a partial clearance of the radiological findings. One patient with disseminated histoplasmosis and typhoid fever died because of multi-organ failure and hypercalcemia [85].

### Univariate and multivariate analyses

Table 3 reports a summary of the univariate and multivariate analyses conducted on all variables of interest. Results from the univariate analysis showed an association between disseminated histoplasmosis and variables such as age (crude OR: 1.03 95% CI: 1.01 to 1.05), Central America (crude OR: 13.01 95% CI: 3.82 to 81.43) or South America (Crude OR: 6.66 95% CI: 1.84 to 42.72) as a destination, and activities that involved inhalation of contaminated soil (crude OR: 3.13 95% CI: 1.72 to 5.62). However, the multivariate logistic regression model did not confirm any association with destination or age, while a strong association was found with activities that involved the exploration of a cave full of bat’s guano (adjusted OR: 34.20 95% CI: 5.29 to 220.93) or other outdoor activities (adjusted OR: 4.61 95% CI: 1.09 to 19.56). No significant difference in the attack rate between countries of destination was observed (*p*-value: 0.8906, Kruskal-Wallis test. Not reported in tables).

**Table 3:** Crude and adjusted ORs for the Association Between Patient’s Characteristics and the Risk of Disseminated Histoplasmosis (DH) among all Patients Recorded in Selected Studies

Covariate ^a^	DH (%) Present	non-DH Absent	Crude OR	Adjusted OR ^b^ (95% CI) ^b^
	56/573 (9.7)	517/573 (90.2)		
Age	29 (22–31)	31 (23–45)	1.03 (1.01–1.05)	1.03 (0.99–1.07)
Male	68.7%	63.4%	1.16 (0.66–2.14)	1.13 (0.37–3.43)
Continent of destination, N (%)				
USA-Canada	3.8%	22.5%	1.00 [Reference]	1.00 [Reference]
Central America	58.5%	26.9%	13.01 (3.82–81.43)	1.28 (0.21–7.67)
South America	30.2%	27.1%	6.66 (1.84–42.72)	1.61 (0.27–9.57)
Africa	5.7%	18.0%	1.87 (0.30–14.45)	2.38 (0.28–20.71)
Others ^c^	1.9%	5.5%	2.05 (0.09–22.18)	0.33 (0.02–4.74)
Activity ^d^, N (%)				
Caves/Bat guano	64.2%	60.5%	1.18 (0.66–2.11)	34.20 (5.29–220.93)
Outdoor activities	42.8%	33.7%	1.48 (0.84–2.57)	4.61 (1.09–19.56)
Bird_chickens	10.7%	19.1%	0.50 (0.19–1.13)	7.19 (0.84–61.57)
Inhalation_soil	37.5%	16.0%	3.13 (1.72–5.62)	2.78 (0.94–8.29)

*Abbreviations*, *OR* odds ratio, *CI* confidence interval

^a^ Relative frequencies are here presented, and the median (interquartile range) is reported for age

^b^ Adjusted for every other covariate through a multivariate logistic regression model

^c^ Includes: Asia, Oceania

^d^ Each activity was entered in the model as a single dummy variable, as most people did more than one activity during the trip. Caves/Bat’s guano: contact with bats excreta and/or visit to a cave. Outdoor activities: forest excursions/trekking, camping or other activities not involving visit to caves or direct contact with bats’ excreta. Bird_chickens: activities involving contact with chickens or other birds. Inhalation_soil: all activities involving building construction, renovation, excavation (biologists, building contractors, construction workers, soldiers, volunteers involved in churches or other buildings renovation)

## Discussion

We were able to identify in the literature 814 cases of histoplasmosis acquired by immunocompetent travelers, for a total of 835 patients including those diagnosed at CTD. Considering that this occurrence is certainly under-diagnosed and under-reported, we assume that the published cases must be the tip of the iceberg. We hope that our systematic review may contribute to raise awareness of the possible occurrence of this condition in travelers visiting endemic countries/areas. It is true that most cases are clinically not severe, nevertheless most of the subjects presented symptoms of various degree, moreover usually long lasting, often implying otherwise unnecessary, invasive diagnostic procedures under the suspicion of more common (and serious) causes such as TB or lung malignancies. Only less than 10% of the subjects were apparently asymptomatic, although it should be recognized the low probability of a pauci- or asymptomatic case to be identified and described in the literature. Of particular note, a relatively large group of patients had Disseminated Histoplasmosis (DH), confirming that this condition, although typical of immunosuppressed individuals, may also occur in immunocompetent subjects. Moreover, given the long course of the infection, an unrecognized/untreated case of histoplasmosis may reactivate later in life, under immune suppressant conditions. Are there any definite risk factors for DH in immunocompetent individuals? We found several associations between disseminated histoplasmosis and other covariates at univariate analysis (Table 2). However, these results appear to be very inconsistent and were not confirmed by multivariate analysis and therefore no conclusion can be drawn from them. At multivariate analysis, the only risk factors resulting significant were exposition to cave-bats and outdoor activities. We found no association between the attack rate and the different endemic areas, hence we cannot suppose that there is a difference in the aggressiveness of the different strains throughout the world. In contrast with this conclusion, some authors [86–90] supposed that different and specific *Histoplasma capsulatum* genotypes could be responsible of the variability of clinical manifestations, in particular some “aggressive” strains might cause a more severe presentation. It is to be mentioned that our result is based on 47 groups only, and the observation of more groups in endemic areas would possibly provide a more robust conclusion. Seventy-eight per cent of reported cases acquired the infection in the American continent, and 57,2% in Central-South America only; these areas should be therefore considered “highly” endemic, although the disease can be seen globally.

From the clinical point of view, when should histoplasmosis be suspected in a returning traveler? Histoplasmosis can be misdiagnosed, not only because of the common low awareness of health-care workers but especially because the clinical picture is mainly characterized by fever (more than 90% of reported cases) associated to respiratory symptoms in around half of cases. More than 80% of patients have a pulmonary form. Therefore, histoplasmosis should be suspected in all travelers presenting with fever/systemic symptoms or community-acquired pneumonia. History should be carefully evaluated, in particular exposure to cave/bats or simply outdoor activities in endemic areas (particularly the American continent) and being part of a cluster of similar illness. Although the most part of cases in our review had also abnormal laboratory exams (two thirds of cases had increased inflammatory markers and half increased transaminases) this is an aspecific finding, especially in pneumonia/febrile systemic illness. However, *Histoplasma* serology is positive in two third of cases. According to Wheat [4], *Histoplasma* galactomannan antigen can be positive in 80–95% of cases in the acute pulmonary and disseminated form and could strongly contribute to diagnosis when available.

Finally, radiologic findings can be of help to distinguish histoplasmosis from bacterial/viral pneumonia in immunocompetent travelers; in our review, 80% of retrieved cases with this information showed pulmonary nodules (typically multiple peripheric nodules with halo sign at computed tomography). Our study has some limitations. First, we excluded some papers by language, in particular those written in Chinese and Dutch, and this could have led to the loss of some cases. We did not find a standardized definition of acute histoplasmosis in the immunocompetent subject in the scientific literature, hence we adapted the definition and the inclusion criteria on the basis of the works of prominent authors, namely Wheat and EORTC/MSG experts [4] [13, 14]. Most of all, it was difficult to retrieve accurate information about the outcome, that was not always described in details. However, with the limitation of a various and not ever reported follow-up, more than 99% of subjects recovered or had a consistent clinical and radiological improvement.

Inconsistency in the analysis of risk factors for Disseminated Histoplasmosis is most probably due to the low quality of the gathered data, that is probably explained by the vast heterogeneity of the sources across the years of publication and the differences in the statistics reported. We tried to implement different logistic models to verify which one could have a better goodness of fit, but unsuccessfully. We also tried data imputation to improve the completeness of the dataset. Namely, we tried to complete data on age and sex through a Multivariate Imputation by Chained Equations (MICE) [88] with the predictive mean matching method, in order to maintain the original distribution of age and sex of the incomplete data. Unfortunately, this did not have an impact on the quality of results. However, the exposure to cave-bats and outdoor activities resulted significant in the majority of models we fitted, so we assume that there is an association with those variables. We finally decided to report results from the best model in terms of goodness of fit.

## Conclusions

Presumably, cases of histoplasmosis in immunocompetent travelers are largely misdiagnosed and under-reported, because these subjects are usually asymptomatic or present mild symptoms. However, it is important to consider this infection in the differential diagnosis in case of epidemiological risk of exposure, also in light of possible (though rare) progression of the disease also in immunocompetent individuals. In addition, the risk of reactivation of the infection in case of subsequent acquired immunosuppression is present, even after a long time [91]. Studies are needed to understand whether antifungal therapy prescribed for the acute, mild form of the disease could prevent further reactivations. Finally, a high index of suspicion might avoid unnecessary hospitalization and invasive procedures.

## Additional files

Additional file 1: Excel sheet including all the demographical, clinical, radiological and treatment characteristics registered for each case. A row of the sheet = a case. (XLSX 412 kb)

Additional file 2: COI disclosure of each Author. (DOCX 90 kb)
