# Supplementary material for: Acute histoplasmosis in immunocompetent travelers: a systematic review of literature
Source: BMC Infect Dis. 2018 Dec 18;18:673. doi: 10.1186/s12879-018-3476-z (PMC6299618; doi:10.1186/s12879-018-3476-z)
Supplement: Supplementary file 2 — COI disclosure of each Author. (DOCX 90 kb) [file 12879_2018_3476_MOESM2_ESM.docx]

|  | **Reference** | **Publication year** | **N° cases** | **Country of origin** | **Visited continent** | **Visited country** | **Reason of travel** | **Exposure** | **Cluster** | **Syndromic classification** | **Therapy** | **Type of therapy** |
| --- | --- | --- | --- | --- | --- | --- | --- | --- | --- | --- | --- | --- |
| 1 | (23) | 1988 | 15 | NS | CA | Costa rica | ST | B | yes | AP | no |  |
| 2 | (24) | 2007 | 4 | Spain | SA | Ecuador | V | C, S | yes | AP | no |  |
| 3 | (25) | 2004 | 1 | France | SA | Colombia | T | B | no | AP | yes | NS |
| 4 | (26) | 2011 | 4 | Italy | SA | Ecuador |  | O | no | AP, DH | 1 yes | Itra |
| 5 | (27) | 1992 | 1 | Germany | SA | Ecuador | T | O | no | AP | no |  |
| 6 | (28) | 2013 | 1 | Israel | SA, A | Jordan, Bolivia, Brazil, Ethiopia, Angola | T | O | no | DH | yes | Itra |
| 7 | (29) | 1995 | 2 | France | SA | Guyana | P | O, S | no | AP | yes | Excision |
| 8 | (30) | 2011 | 5 | Spain | A, SA, CA | Angola, Venezuela, Nicaragua, Ecuador | T, P, SP, V | B, O | no | AP | no |  |
| 9 | (31) | 2002 | 14 | Canada | CA | Belize | ST | O | yes | AP | 1 yes | itra |
| 10 | (32) | 2013 | 1 | USA | SA | Peru | V | B, S | no | DH | yes | AmB, itra |
| 11 | (33) | 1999 | 1 | France | SA | Guyana | P | O, S | no | DH | yes | itra |
| 12 | (34) | 2013 | 12 | Europe, Africa | A | Uganda | ST | O | yes | AP | 5 yes | 4 itra, 1 keto |
| 13 | (35) | 1979 | 10 |  | A | South Africa | SP | B | yes | 9 AP, 1 DH | 1 yes | AmB |
| 14 | (36) | 1997 | 1 | France | SA | Guyana | P | O, S | no | AP | yes | Itra |
| 15 | (37) | 2006 | 1 | Netherland | A | Ghana | T | B, O | no | AP | no |  |
| 16 | (38) | 1957 | 2 | South africa | A | South Africa | SP | B | yes | AP | no |  |
| 17 | (39) | 2012 | 1 | Germany | SA | Costa Rica | T | B, O | no | AP | yes | itra |
| 18 | (40) | 2002 | 6 | Germany | CA | Cuba | BIO | B, S | yes | 3 AP, 3 DH | yes | itra |
| 19 | (41) | 2000 | 4 | Italy | CA, SA | Guatemala, Dominican republic, Peru | T, SP | B, O, S | no | 3 AP, 1 DH | yes | 2keto, 1 itra, 1 amB |
| 20 | (42) | 2005 | 1 | Italy | CA | Nicaragua | P | O, S | no | AP | yes | itra |
| 21 | (43) | 2003 | 3 | Spain | CA | Nicaragua | T | B, O | yes | AP | 1 yes | itra |
| 22 | (44) | 1988 | 11 | USA | USA | Iowa | T | O, S | yes | AP | no |  |
| 23 | (45) | 2005 | 9 | Spain | SA | Guatemala | V | S | yes | AP | no |  |
| 24 | (46) | 2000 | 7 | Spain | CA, SA | Dominican Republic, Nicaragua, Colombia, Peru, Guatemala | T, V | B, O, S | no | 1 AP, 8 DH | yes | itra |
| 25 | (47) | 1962 | 3 | Europe | A | South Africa | SP | B | yes | DH | yes | AmB |
| 26 | (48) | 1990 | 1 | France | SA | Guyana | T | O | no | AP | no |  |
| 27 | (49) | 1981 | 69 | USA | NA | South Carolina | CI | C | yes | AP | no |  |
| 28 | (50) | 1975 | 6 | Canada | CA | Puerto Rico | ST, TEA | B, O | yes | AP | no |  |
| 29 | (51) | 1979 | 1 | Jamaica | CA | Caribbean | TEA | B | no | AP | no |  |
| 30 | (52) | 1996 | 1 | Germany | CA |  | T | O | no | AP | no |  |
| 31 | (53) | 2008 | 3 | Austria |  | Mexico | T | B | no | DH | yes | itra |
| 32 | (54) | 2008 | 3 | Germany | CA | Antille | BIO | B, S | yes | 2 DH, 1 AP | yes | itra |
| 33 | (55) | 2012 | 4 | Poland | SA | Ecuador | T | B | yes | DH | 2 yes | keto |
| 34 |  | 2003 | 1 | Germany |  | Mexico, Brazil | T | O | no | AP | no |  |
| 35 | (7) | 2015 | 1 | Swiss | SA | Brazil, Argentina |  | C | no | AP | yes | itra |
| 36 | (16) | 1999 | 1 | Taiwan | SEA | Indonesia | P | O | no | DH | yes | AmB |
| 37 | (56) | 2011 | 2 | France | CA | Costa Rica | T | O | yes | AP | 1 yes | 1 itra |
| 38 | (57) | 1979 | 27 | USA | USA | North-Centre Florida | V | B, O, S | yes | 8 As, 2 DH  17 AP | 2 yes | 2 AmB |
| 39 | (58) | 2004 | 10 | USA | CA | Costa Rica | T | B | yes | AP | no |  |
| 40 | (59) | 2013 | 1 | Canada | CA | Costa Rica |  | B, O, S | no | DH | yes | AmB, itra |
| 41 | (60) | 2005 | 2 | France | SA | Venezuela | P | O, S | no | AP | yes | itra |
| 42 | (61) | 1991 | 1 | France | SA | Guatemala | T | O, S | no | AP | yes | keto |
| 43 | (15) | 2003 | 262 | USA |  | Mexico | T | O, S | yes | AP | yes | NK |
| 44 | (62) | 2014 | 1 | Germany | SA | Ecuador | T | O | no | AP | yes | itra |
| 45 | (63) | 1957 | 64 | Europe, NS | A | South Africa | ST, SP | B | yes | AP, 5 As | no |  |
| 46 | (64) | 1997 | 4 | Italy | SA | Peru | SP | B, C | yes | DH | yes | keto |
| 47 | (65) | 1995 | 24 | Europe, SEA | AU | New Caledonia | SP | B | yes | AP, 3 As | 21 yes | Keto, itra, AmB |
| 48 | (66) | 2005 | 5 | Spain | CA, SA | Peru, Costa Rica, El Salvador, Panama, Ecuador | T, BIO, V, P | B, C, O, S | no | 3 AP, 2 DH | Yes 1 pulmonary and 2 DH | itra |
| 49 | (67) | 2010 | 3 | Japan |  | Malaysia | T | O | yes | AP | no |  |
| 50 | (68) | 2001 | 1 | Spain | SA | Peru | T | B, O | no | AP | yes | itra |
| 51 | (69) | 2014 | 4 | Brazil | SA | Brazil | BIO | B | yes | AP | 2 yes | itra |
| 52 | (70) | 1986 | 6 | USA | USA | Florida | ST, P | B, S | yes | AP | 4 yes | 2Amb,2keto |
| 53 | (71) | 2006 | 1 | USA | CA | Guatemala | T | O | no | R | yes | itra |
| 54 | (72) | 2003 | 13 | France | CA | Martinica | Trekking trip | O, S | yes | 11 DH, 2 AP | yes | itra |
| 55 | (73) | 2009 | 1 | Germany | CA | Antille | BIO | B, S | no | AP | yes | NS |
| 56 | (74) | 2015 | 23 | Israel | USA, CA, SA | Guatemala, Costa Rica Peru, Mexico, Bolivia, Indiana, Dominican Republic | NS | B, O | no | 14 AP, 9 As | Not known |  |
| 57 | (75) | 2011 | 3 | France | CA | Cuba | SP | B | yes | 1 DH, 2 AP | yes | itra |
| 58 | (76) | 2007 | 1 | Japan | SA | Bolivia | P | O, S | no | AP | yes | fluco |
| 59 | (77) | 1992 | 1 | Italy | A |  | P | O, S | no | AP | yes | AmB |
| 60 | (78) | 2000 | 12 | Spain | CA, SA | Guatemala, Honduras, Nicaragua, Dominican Republic, Peru | V, SP, T | B, O, S | yes | 7 AP, 5 As | 8 yes | itra |
| 61 | (79) | 1999 | 11 | USA | SA | Ecuador | ST | B | yes | 1 DH, 8 AP, 2 not possible | 4 yes | 1 itra, 3 systemic azoles |
| 62 | (80) | 2008 | 20 | USA | CA | El Salvador | V | O, S | yes | AP | Not known |  |
| 63 | (81) | 2003 | 14 | USA | CA | Nicaragua | ST | B | yes | 4 DH, 8 AP, 2 As | yes | itra |
| 64 | (82) | 1999 | 6 | Chile | SA | Ecuador | T | C, O | yes | AP | yes | itra |
| 65 | (83) | 1966 | 2 | USA | USA | Missouri, Kentucky | T | C | no | AP | 1 yes | AmB |
| 66 | (84) | 2015 | 1 | USA | USA | Southwest | T | O, S | no | AP | yes | itra |
| 67 | (85) | 2010 | 4 | Brasil | SA | Brasil | SP | B | yes | 2AP, 2DH | yes |  |
| 68 | (86) | 2012 | 5 | Argentina | SA | Argentina | O | C | yes | AP | no |  |
| 69 | (87) | 2002 | 1 | Chile | SA | Peru | SP | B | no | AP | Yes |  |
| 70 | (88) | 2001 | 31 | Venezuela | SA | Venezuela | SP | B | yes | AP | No |  |
| 71 | (89) | 1981 | 33 | Colombia | SA | Colombia | SP | B | yes | AP | No |  |
|  | CTD Negrar | 2016 | 21 | Italy | SA, CA | Ecuador, Bolivia, Mexico, Cuba | T, SP, P | B, O, S | 16 yes, 5 no | 1 DH, 20 AP | 11 yes | itra |

Table 3: Studies selected for the systematic review.

NS: not specified. USA: United States of America. NA: North America. CA: Central America. SA: South America. A: Africa. AU: Australia. SEA: South East Asia. ST: student. V: Volunteer. T: Tourist. P: professional. SP: Speleologist. BIO: Biologist. CI: Correctional Institute. TEA: Teacher. B: Cave_bats. C: Birds/chickens. S: Moved Soil. O: Outdoor activities. DH: Disseminated Histoplasmosis. AP: Acute Pulmonary Histoplasmosis. AmB: Amphotericin B. Itra: Itraconazole. Fluco: Fluconazole. Keto: Ketoconazole.
